# Catalina Devandas Aguilar: empowering people with disabilities

**DOI:** 10.2471/BLT.19.030119

**Published:** 2019-01-01

## Abstract

Catalina Devandas Aguilar talks to Stephanie Cheng about the impact of the 2008 *Convention on the Rights of Persons with Disabilities,* the importance of listening to people living with disabilities and what United Nations agencies can do to support their further empowerment.

Q: What led you to work in advocacy for people with disabilities?

A: I was working on women’s rights as a lawyer in Costa Rica and was invited to a meeting of an organization working for people with disabilities in 1999. That meeting got me focused on the particular needs of that group from a rights perspective, something that I hadn’t really considered before and also started me thinking about advocacy. I went on to work with national advocacy organizations in Costa Rica and then onto their regional equivalents. In 2004 I went to work with the World Bank as a consultant for the Disability and Inclusive Development team for the Latin America and the Caribbean region.

Q: You participated in the negotiation of the Convention on the Rights of Persons with Disabilities. What was that like?

A: It was a life-changing experience. Anyone that participated in the process can attest to the level of intensity and the incredible atmosphere of the negotiations. It was amazing to witness the active participation of people with disabilities and their organizations in interaction with diplomatic delegations at the highest level in the United Nations. I learnt so much, for example, how to frame issues and manage negotiations in a way that advances an agenda. When I returned to working with grassroots disabilities organizations, I was in a better position to contribute because I could navigate both the international and grassroots advocacy. I felt that my experience could help bridge the gaps between these two worlds.

Q: How have your experiences fed into your work as Special Rapporteur?

A: My grassroots advocacy work and personal experiences of disability make me focus on the practical, day-to-day challenges faced by people.

Q: Could you talk about the main health-related challenges that people with disabilities face?

A: There are so many of them. To start with, people with disabilities don’t have basic access to health care. In many places there is no universal health coverage and private health insurance companies refuse to cover them. In some places, people with disabilities cannot even access health centres. The barriers are not only physical of course. Misconceptions about people with disabilities are an important problem and this affects their interaction with health systems. Take for instance the issue of informed consent. In many cases it is wrongly assumed that people with disabilities cannot consent to medical interventions, sometimes leading to health workers administering treatment against the will of the person concerned. Unfortunately, many countries have mental health legislation that allows for involuntary treatment. In the case of women with disabilities, involuntary sterilizations are a grave concern. I have not found any country that has legislation that forbids these operations. There is a need for change. The World Health Organization (WHO) should recommend abolishing this kind of legislation, recognizing that the rights of people with disabilities are the same as the rights of everyone else.

“I actually took the handles off my wheelchair to stop people pushing me.”

Q: What about challenges outside health systems? Can you talk about those?

A: Once again, there are many, most of them relating to different forms of social or economic exclusion. To focus on just one aspect, excluding people with disabilities from the workforce affects their income and their self-esteem. Here too change is needed, and not just in terms of the kind of financial support countries offer. Instead of saying, “OK, we have social protection and we will give you a pension and that’s it, you’re out of poverty,” our aim should be broader. People with disabilities should of course benefit from social protection schemes and health insurance, but they also need to be able to participate fully in the world of work and in society generally. They do not want to be kept out, isolated at home. They want to live their lives to the full, just like anyone else. They have a great deal to contribute.

Q: What difference would you like to make as Special Rapporteur?

A: My ultimate goal as Special Rapporteur is to shift perceptions, so that people start to see different functional abilities and ways of existing as part of the spectrum of human diversity, rather than something that you have to cure or correct. Changing attitudes towards people with disabilities needs to be a priority, considering us as a normal part of society, as fully fledged citizens and not a subgroup requiring pity. We need respect and that starts with listening to us. I’ll give you a concrete example. You might think that by pushing someone in a wheelchair that you are helping them and that might be the case, but you need to ask, “Do you need help?” If you push someone’s chair without taking into consideration the person’s view, you are not really helping, you’re taking away their control. I actually took the handles off my wheelchair to stop people pushing me. This simple example also illustrates how people with disabilities are treated in every single aspect of their lives. Of course, it is very difficult to change perceptions. Deeply entrenched attitudes are hard to change. People still think that people with disabilities are sick and should be “cured,” or that you are a superhero if you have a disability and are living a normal life. Because I am in a wheelchair people say to me: “Oh you have a family! Wow!” It makes me laugh. Why wouldn’t I have a family? I am a mother of three girls.

Q: You sound a little pessimistic.

A: Not at all. I am optimistic that things can change, and I see that attitudes are improving. It varies from country to country, but these changes are taking place, and I think the *Convention on the Rights of Persons with Disabilities* has made an immense difference. Sometimes people think, particularly at the grassroots levels, that the discussions we are having at an international level have no effect on the realities and the lives of people on the ground, but that is not the case. These discussions have a significant impact.

Q: What can WHO do to help change attitudes?

A: WHO and other United Nations agencies can play an important role in encouraging people to reflect and change the way they think about disability, starting with the way they discuss and write about the issue. WHO has been an incredible partner to people with disabilities for a long time, but it’s important to recognize that we now have new perspectives, partly because of the convention, that need to be incorporated in the discourse of WHO and the UN system. But other things need to change too, notably legislation that disempowers people with disabilities; there are already signs of progress here, which is very encouraging. 

When we were negotiating the convention, we weren’t sure we were going to get changes in the area of legal capacity. People were saying that we were completely unrealistic; it was not going to happen. Now you have more than 30 countries that have changed their legislation to prohibit people taking decisions on behalf of persons with disabilities without that person’s consent. It is also important to prioritize this issue in the global public health agenda, so that it gets the visibility it merits. 

The fact that the sustainable development goals make several references to people with disabilities is an important first step. Greater visibility for the issue of disability will also come with better information. Here too, there has been some progress with the main international donors, beginning to use indicators to measure the extent to which people with disabilities are included in different programmes.

“It is not necessarily a question of spending more, but rather of taking into account different disability issues when designing programmes.”

Q: What would you like to have achieved by the end of your mandate?

A: I would like the United Nations system to fully embrace the *Convention on the Rights of Persons with Disabilities,* and to try to have a United Nations system-wide approach to people with disabilities. For example, with regard to procurement, how are United Nations agencies purchasing goods? Are those goods accessible? Is everything the United Nations rents, owns or builds accessible to people with disabilities? Regarding programme design and development, are the needs of people living with disabilities reflected? It is not necessarily a question of spending more, but rather of taking into account different disability issues when designing programmes. 

It would also help if people with disabilities were consulted in the planning of international health programmes. This means making sure that information about those programmes is accessible and that people with disabilities are aware of them and encouraged to participate in any discussions related to those programmes.

Q: Can you give us an example?

A: For instance, when designing international programmes for deaf people, you have to ask how you are getting this information to deaf people. Do you have interpreters? Are you actually consulting the people you are trying to reach with your programmes? I am working closely with all United Nations entities to support the decisions made by the Secretary General to adopt a system-wide approach to disabilities issues, which we hope will be fully implemented one day. There also needs to be an accountability mechanism for the plan to be more than just an aspirational document. If I achieve all that by the end of my mandate in two years, I will be happy.

